# Indents

**Published:** 1903-05-15

**Authors:** 


### INDENTS.

☞ **Brief Articles of Technical or Scientific Value will receive notice and comment. Contributions invited.**

**Evolution of Pus.** Where pus has been diagnosed in alveolar abscess, a good way to draw it off is by the use of an aspirating syringe.—Ed. *Cosmos*.

**To Clean Impression Trays.** Remove the wax by heating and wiping with paper or cloth, then boil in a solution of washing soda.—J. R. Watts, in *Review*.

**Formaldehyde as a Disinfectant.** A ten-percent solution will sterilize instruments in from ten to twenty minutes, and it makes a good deodorant used in the cuspidore.—*Dominion Journal*.

**Erasing Powder.** Alum, sulphur, powdered pumice and potassium nitrate, equal parts, well pulverrized and triturated together, make a good powder to erase recent writing or ink blots. Apply with a clean cloth. Keep in a well-corked bottle.—*Western Druggist*.

**Replacing a Broken Crown.** When it is impracticable to remove the the pin from the root, run a drill or trephine around the pin, make a tube of platinum to fit the post and solder to cap as for a Richmond crown, proceeding as for any other crown.—E. E. Cruzen, *Dental Cosmos*.

**Amalgam Fillings.** Don't use the same mix of amalgam for the whole of a large cavity, or the filling will be softer on the surface than beneath, thus inducing a shrinkage that will destroy the adaptation of the filling to the cavity margin.—Harold Clark, *Dom. Dental Journal*.

**Investment for Inlay Work.** A bottle of powdered asbestos and water of creamy consistency can be kept in the cabinet, near the chair, ready for use at any moment, and will be found much cleaner and neater to handle than mixing for each case.—F. T. Van Woert, *Dental Summary.*

**Oxyphosphate for Inlay Impressions.** Dust the cavity with pulverized soapstone and insert a pellet of quick-setting oxyphosphate, letting it extend over the edges of the cavity. When set remove, dust the surface with soapstone, and make a mold of the same material.—*Dental Cosmos.*

**Chronic Alveolar Abscess.** Cleanse the root-canal and insert a dressing of cotton saturated with a saturated alcoholic solution of thymol crystals. Seal tight with gutta-percha and leave in place for four or five days. Repeat if necessary.—W. H. Hersh, *Dental Review.*

**Poisoning from the External Use of Chromic Acid.** Several cases of profound toxic symptoms (going on to death, it is believed by some), have resulted from the external use of chromic acid solution. No doubt these cases were idiosyncratic, yet the fact remains and should be heeded.—*Monthly Cyclopædia.*

**Pulp Extirpation with Cocain.** Dissolve cocain crystals in adrenalin and there will be no hemorrhage. Wrap a few fibers of cotton on a broach and dip in trichloracetic acid and insert to the bottom of the canal, cauterizing the ends of the nerve fibers. The canal is then ready for immediate filling.—A. Enbank, *Dental Headlight.*

**To Clean Broaches and Clamps.** Fill a wide-mouth tooth-powder bottle half full of a ten-percent solution of Lysal and add a teaspoonful of fine sand. Place the broaches and clamps or burs in the solution, cork the bottle and shake and let stand ten minutes and shake again as often as may be necessary. Then remove and rinse in clean water to which a little listerine or soda has been added.

**Antidote for Phenol.** Alcohol is the best agent for counteracting the escharotic tendency of carbolic acid. Alcohol has a strong affinity for phenol and prevents its acting upon the albumens as an escharotic. Alcoholic solutions of phenol are not cauterant. In case of accidental applications of phenol to the gums or lips, apply alcohol at once and it will check the threatened cauterant action.—*Office and Laboratory.*

**Wine of Opium.** This preparation has been recommended forcdontalgia, for aching pulps and gum tissues. It has been especially serviceable to me in relieving pain of pulpless teeth which continue to ache after local conditions have been made favorable by instrumental efforts. Applied in the pulp canal it produces a desirable soothing influence on the apical tissues.—J. M. Howe, in *International Journal.*

**Mentho-Phenol.** Made by melting three parts of menthol and one part of phenol crystals together, as an obtundent antiseptic dressing in root-canals where there is an irritable condition of the apical structures, is a valuable remedy. It is also a good remedy to apply to an aching pulp or to painful conditions of a tooth socket after extracting, for its anodyne properties.—J. M. Howe, in *International Journal.*

**Cocain Solution for Pyorrhoea Work.** To lessen the pain in cleansing the teeth in pyorrhœa work, make a syrupy solution of phenol and cocain. Saturate a rope of cotton with the solution and pack it into the pockets for five minutes before operating. The phenol prevents the absorption of the cocain and the cocain overcomes the cauterant action of the phenol. After the scaling apply a stimulating irrigant or lotion.—C. F. W. Bodecker, in *Review.*

**To Remove Silver Stains from the Fingers.** Dip the fingers into a mixture of copper sulphate eight parts, and hydrochloric acid two parts, and water ninety parts. Then wash them in a sodium hyposulphate solution, using plenty of friction, and lastly rinse in clean water.—*Western Druggist.* Silver stains may also be removed by saturating them with iodine, which converts the silver salts into iodide of silver, which is soluble in a saturated solution of sodium hyposulphate. —Ed. *Cosmos.*

**Position of the Molar Teeth.** In artificial teeth the grinding surfaces should not meet laterally on a horizontal plane; there should be a decided incline of the surface of these teeth from the buccal to lingual cusps. This makes the upper teeth to fit into the lower as a ball and socket joint and tends by directing the pressure against both alveolar arches to make plates set steadily, as the teeth are moved upon each other in mastication. It accomplishes the same object in lateral movement that the antero-posterior occlusal line does for this movement in mastication.—J. A. Robinson, in the *International Journal.*

**Waxing Up Artificial Teeth Made Easy.** Take the bottom of a greased tin box, put your old scraps of base-plate wax in it, melt them up hot, then paint on the wax with an artist's paint-brush one-quarter inch wide. Keep wax hot, and laugh to think how easily you can do it. I do not know but that this is old, but it is original on my part—never saw anything in print or ever heard any one mention the thing. If it is new score me one, for it is the first original idea I ever had.—E. C. French, *Amer. Dent. Jour.*

**Pulp Extirpation.** When you have removed a pulp, if a particle of nerve is left the electric current will find it out. The induction current can be applied by using a little dry battery, such as in one of the "ever-ready" lamps, using the secondary coiled pulled out to its fullest degree and gradually pushed in until response is had. Solder to one end of a piece of German silver wire a little tube to hold a little cotton; wet the cotton and apply it to the tooth, the negative being held in the patient's hand. It is a better test, in pulp diagnosis, than heat, cold or light.—Dr. Greevert, *Dental Cosmos.*

**Cause of Breaking of a Vulcanite Denture.** Another fertile cause of the breaking of a vulcanite denture is the not recognizing the fact that each natural tooth on the model makes an indentation in the rubber. Now, if one when molding the wax to such a model should get it attenuated or thinned over the convexity represented by the tooth, then one has a weak spot that does not require much of a strain to snap the case in halves; so that too much care cannot be exercised in keeping the contour of the model, so that the rubber plate may present as nearly as possible an even thickness.—Harry Rose, *Journ. Brit. Den. Asso.*

**Tooth Corner Restored.** Cut the surface smooth with a diamond disk and then excavate a cavity without undercut. Burnish into this cavity a platinum matrix, and bake it full of porcelain. Select a plate tooth of the proper form and color, and cut off of it enough of its corresponding corner to restore the corner as desired. Grind to fit and wax the matrix containing the porcelain. Mark so the parts may be reassembled, and separate so that the parts in contact may be ground out hollow. Then fill this cavity between the two pieces with a low-fusing body and fuse together in the furnace. The piece may then be set in place with cement and held firmly until the cement has hardened.—Dr. L. F, Jack, in *Cosmos.*

**Extracting Roots Without Destroying the Process.** I noticed in the questions of the January number advice as to the kinds of forceps for removing badly-decayed roots and teeth. I found a better method suggested to me by having a very badly-broken lateral incisor in the mouth of a hospital patient. If I had crushed the process I would have left a deformity, so I anesthetized the gum, took a long fissure bur and passed it well around the root, removing any process, then took a small forceps and removed the remaining root without any trouble whatever. I have had success in a number of cases by using this method. This mode can be used anywhere in the mouth, especially when you want to preserve the alveolar process.—Frances Gregory Crouch in *Dental Brief*.

**Thorough Malleting of Contact-Point in Approximal Gold Fillings.** In gold fillings the contact point should receive more than the ordinary amount of malleting, and in this connection it may be stated that if for no other reason than this a matrix is indicated in filling thiese cavities when they occur on the distal surfaces of the teeth. With a thin matrix lying between the filling and the approximal tooth, the gold may be solidly driven against the contact point of the tooth without injury to the enamel on account of the protection afforded it by the matrix, and when the filling has been properly contoured and hardened and the matrix removed, the gold will be found in perfect contact with the approximal tooth, and with very little finishing necessary to make a good surface.—C. N. Johnson, *Dom. Dent. Jour.*

**Cavity Varnishes.** We are frequently called on to fill cavities which, owing to their shallowness, their sensitiveness, or the fear of exposure of the pulp, are difficult to so shape that the first few pieces of gold can be easily packed so as to remain in place until the filling is anchored. In such cases a preparation of the kind mentioned is of much assistance. In, fact it has several uses, such as assisting to keep wooden wedges in place when used to hold teeth apart while being filled; these wedges have a tendency to slip out of place, which can be prevented by wetting the wedge with the varnish before inserting it. It is also valuable to hold the rubber dam in place, especially in such cases where we wish to avoid the use of a ligature. It is also advantageously employed in preventing the thermal shock so often complained of by patients after a tooth has been filled with gold or amalgam, also in the case of devitalized teeth filled with amalgam, it to some extent, prevents the discoloration so often caused by that material.—Dr. Wessels in *Brief*.

**Some Physical Principles Applied in Dentistry.**

Capillary attraction is but another form of adhesion, and has many practical applications in dentistry. It is this force that enables us to separate teeth by the use of cotton, tape, cardboard, etc., packed or drawn between them. By capillary attraction the fluids are taken up by these substances, forcing the fibers apart and gradually exerting a pressure sufficient to move the teeth in their sockets. To the writer, one of the most valuable and satisfactory applications of this principle is in the application of medicinal agents to root-canals in the treatment of pulpless teeth. When it is desired to flood the canal with the medicament, to insure its reaching the apex of the root, a broach as nearly the size of the canal as possible is passed to the apex in the empty canal. By dipping the point of the closed foil pliers into the agent to be used, a sufficient quantity will be retained between the beaks by capillary attraction, and when the points of the pliers are placed at the opening of the canal into which the broach has been passed and the pliers allowed to open slowly, the fluid is deposited at the mouth of the canal and by capillary attraction, when the broach is gently worked laterally, is passed between the walls of the canal to the apex. The broach serves the double purpose of displacing the air in the canal and aiding the capillary force, thus insuring the passage of the fluid to the apex.—N. C. Leonard, *Dental Headlight*.

